# Insights into the protonation state and spin structure for the *g* = 2 multiline electron paramagnetic resonance signal of the oxygen-evolving complex

**DOI:** 10.1093/pnasnexus/pgad244

**Published:** 2023-07-28

**Authors:** Keisuke Saito, Shunya Nishio, Mizue Asada, Hiroyuki Mino, Hiroshi Ishikita

**Affiliations:** Department of Applied Chemistry, The University of Tokyo, 7-3-1 Hongo, Bunkyo-ku, Tokyo 113-8654, Japan; Research Center for Advanced Science and Technology, The University of Tokyo, 4-6-1 Komaba, Meguro-ku, Tokyo 153-8904, Japan; Department of Applied Chemistry, The University of Tokyo, 7-3-1 Hongo, Bunkyo-ku, Tokyo 113-8654, Japan; Instrument Center, Institute for Molecular Science, 38 Nishigo-Naka, Myodaiji, Okazaki 444-8585, Japan; Division of Material Science, Graduate School of Science, Nagoya University, Furo-cho, Chikusa-ku, 464-8602 Nagoya, Aichi, Japan; Department of Applied Chemistry, The University of Tokyo, 7-3-1 Hongo, Bunkyo-ku, Tokyo 113-8654, Japan; Research Center for Advanced Science and Technology, The University of Tokyo, 4-6-1 Komaba, Meguro-ku, Tokyo 153-8904, Japan

## Abstract

In photosystem II (PSII), one-electron oxidation of the most stable oxidation state of the Mn_4_CaO_5_ cluster (S_1_) leads to formation of two distinct states, the open-cubane S_2_ conformation [Mn1(III)Mn2(IV)Mn3(IV)Mn4(IV)] with low spin and the closed-cubane S_2_ conformation [Mn1(IV)Mn2(IV)Mn3(IV)Mn4(III)] with high spin. In electron paramagnetic resonance (EPR) spectroscopy, the open-cubane S_2_ conformation exhibits a *g* = 2 multiline signal. However, its protonation state remains unclear. Here, we investigated the protonation state of the open-cubane S_2_ conformation by calculating exchange couplings in the presence of the PSII protein environment and simulating the pulsed electron–electron double resonance (PELDOR). When a ligand water molecule, which forms an H-bond with D1-Asp61 (W1), is deprotonated at dangling Mn4(IV), the first-exited energy (34 cm^−1^) in manifold spin excited states aligns with the observed value in temperature-dependent pulsed EPR analyses, and the PELDOR signal is best reproduced. Consequently, the *g* = 2 multiline signal observed in EPR corresponds to the open-cubane S_2_ conformation with the deprotonated W1 (OH^−^).

**Significance Statement**

Photosystem II (PSII) is a protein complex that catalyzes the light-driven oxidation of water to molecular oxygen. We investigated the protonation state of the oxygen-evolving Mn_4_CaO_5_ cluster in PSII using a quantum mechanical/molecular mechanical approach that fully considers the protein environment, including the H-bond network. By combining with pulsed electron–electron double resonance simulations, we identified the open-cubane S_2_ conformation with the ligand water molecule (W1) deprotonated at the dangling Mn4(IV) site as the source of the *g* = 2 multiline signal observed in electron paramagnetic resonance spectroscopy. The results demonstrate the importance of considering the protein environment in interpreting spectroscopic data and suggest alternative methods to investigate the protonation state and spin structure of the metal complex.

## Introduction

**Fig. 1.**
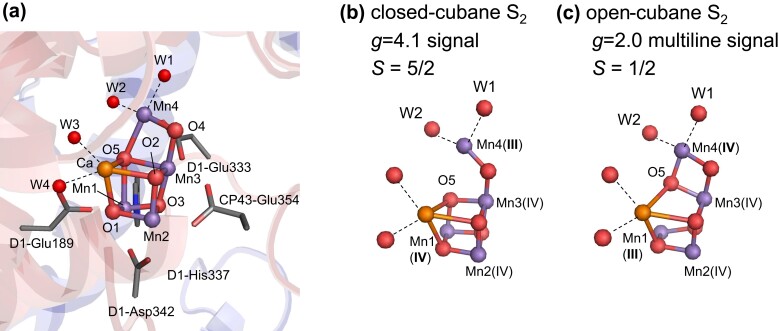
Mn_4_CaO_5_ cluster in PSII. (A) Protein environment of the Mn_4_CaO_5_ cluster. (B) Closed-cubane S_2_ conformation (Mn1(IV) and Mn4(III)) with W1 = OH^−^ and W2 = H_2_O (3). (C) Open-cubane S_2_ conformation (Mn1(III) and Mn4(IV)). Dotted lines indicate ligand interactions.

The Mn_4_CaO_5_ cluster in photosystem II (PSII) plays a vital role as the catalytic center for oxidizing substrate water molecules (1, 2). The oxidation state of the Mn_4_CaO_5_ cluster, denoted as *S_n_* (*n* = 0, 1, 2, or 3), increases with electron transfer (Fig. 1), leading to O_2_ evolution during the S_3_ to S_0_ transition. As the reaction progresses, protons are released in the S_0_ → S_1_ → S_2_ → S_3_ → S_0_ transitions with a stoichiometry of 1:0:1:2. The Mn_4_CaO_5_ cluster consists of three Mn and one Ca sites in the cubane region (Mn1, Mn2, Mn3, and Ca) and a dangling Mn site (Mn4) (Fig. 1). Two ligand water molecules, W1 and W2, are present at the Mn4 site, while two additional water molecules, W3 and W4, are located at the Ca site. In the high oxidation state model for S_1_ (3), the Mn valence state is Mn(III)_2_Mn(IV)_2_ and Mn2 and Mn3 are already oxidized to Mn(IV) based on the redox potential (4). Thus, either Mn1(III) or Mn4(III) serves as the oxidation site in the S_1_ to S_2_ transition.

The closed-cubane S_2_ conformation [Mn1(IV)Mn2(IV)Mn3(IV)Mn4(III)] forms (Fig. 1A), as the Mn1(IV)–O5 bond shortens and the Mn4(III)–O5 bond lengthens upon the oxidation of Mn1(III) to Mn1(IV) during the S_1_ to S_2_ transition (5, 6). Conversely, the open-cubane S_2_ conformation [Mn1(III)Mn2(IV)Mn3(IV)Mn4(IV)] forms (Fig. 1C), as the Mn1(III)–O5 bond lengthens and the Mn4(IV)–O5 bond shortens upon the oxidation of Mn4(III) to Mn4(IV) during the S_1_ to S_2_ transition (5, 6). The open-cubane S_2_ conformation has been observed in the X-ray free electron laser (XFEL) structures, whereas the closed-cubane S_2_ conformation has not been identified (7–10). This observation suggests that the open-cubane S_2_ conformation is more energetically favorable than the closed-cubane structure in cyanobacterial PSII (11–14).

In the S_2_ to S_3_ transition, a water molecule is incorporated into the Mn_4_CaO_5_ cluster according to the XFEL structures (7–10). This transition also involves a two-step proton transfer process: the release of the proton from the Mn_4_CaO_5_ cluster and the transient protonation of D1-Asp61 (15–18), followed by subsequent proton transfer via D1-Glu65/D2-Glu312 toward the lumenal bulk surface (19). The experimentally observed small (∼1) and large (∼2) kinetic isotope effects (20) likely correspond to the first and second processes, respectively.

Electron paramagnetic resonance (EPR) spectroscopy is a method to determine the spin structure and the protonation state of the Mn_4_CaO_5_ cluster and W1–W4. In EPR spectroscopy, two signals are observed as follows: the *g* = 2 multiline signal and the *g* ≥ 4.1 signals (e.g. (21–25)). The *g* ≥ 4.1 signals can be categorized into two groups as follows: the *g* = 4.1 signal and the *g* ∼ 5 signal (26, 27). The *g* = 4.1 signal corresponds to the high-spin closed-cubane S_2_ conformation (5, 6, 28), while the *g* ∼ 5 signal geometry has not been identified yet. To determine the protonation state of the Mn_4_CaO_5_ cluster and the ligand water molecules (W1–W4), results were usually interpreted using quantum chemical calculations of the high-spin S_2_ conformation conducted mostly in the absence of the PSII protein environment for simplicity (5, 6, 29, 30). These theoretical models with the isolated Mn_4_CaO_5_ cluster proposed that W1 = H_2_O and W2 = OH^−^ (5, 6, 30). In contrast, recent theoretical studies conducted in the presence of the PSII protein environment indicated that W1 = OH^−^ and W2 = H_2_O for the *g* = 4.1 signal, as the *g* = 4.1 EPR signal was reproduced only when the high-spin closed-cubane S_2_ conformation (total spin ***S*** = 5/2) had W1 = OH^−^ and W2 = H_2_O, not W1 = H_2_O and W2 = OH^−^ (Fig. 1B) (28). The *g* = 4.1 signal was observed in plant PSII, but not in cyanobacterial PSII under physiological conditions (31, 32). This observation aligns with the energetically unstable nature of the closed-cubane S_2_ conformation in cyanobacterial PSII (28). Thus, it is a prerequisite to consider the protein environment in theoretical calculations when interpreting EPR spectroscopy (28).

On the other hand, the *g* = 2 multiline signal corresponds to the open-cubane S_2_ conformation with low spin (***S*** = 1/2) (5, 6). *T*_1_ (electron spin-lattice relaxation time) measurements indicated that an excited spin state manifold exists 22–37 cm^−1^ above the ground state manifold corresponding to the *g* = 2 multiline signal (33, 34). The ^55^Mn electron nuclear double resonance (ENDOR) analysis was used to probe the hyperfine interaction (HFI) constants (e.g. the isotropic part of the effective HFI constant *A*_iso_) of Mn sites in the S_2_ low-spin state (35, 36). In ENDOR experiments, the HFI constants of the four Mn sites were obtained as the set of four values for *A*_iso_ of −245, 217, −297, and 200 MHz (37) or 312, 251, 208, and 191 MHz (29) for the four Mn sites.

Pantazis et al. converted spin densities into HFI constants using the following equation:


(1) $$A_{i,\mathrm{iso}}=a_{i,\mathrm{iso}}\;\rho_{i},$$

where *A_i,_*_iso_ is the isotopic part of the HFI constant for the *i*-th Mn site (Mn(*i*), where *i* = 1, 2, 3, and 4), *a_i,_*_iso_ is the intrinsic HFI constant, and *ρ_i_* is the spin projection (36). Importantly, the equation is *guaranteed only in the absence of the zero-field splitting* (ZFS), i.e. the anisotropy of the spin projection matrices *ρ****_i_*** can be neglected and *ρ****_i_*** are proportional to the identity matrix (36). Although Eq. 1 is applicable to the model compounds of a two-spin system (38), it remains unclear whether the Mn_4_CaO_5_ cluster is the case.

Using Eq. 1, the *A*_iso_ values in the low-spin S_2_ state were calculated in the absence of the PSII protein environment with density functional theory (DFT) methods, e.g. (−276, 170, 165, and −228 MHz) (5) and (342, 245, 207, and 195 MHz) (34) for the four Mn sites. However, the assignment of *A*_iso_ to Mn sites did not fit quantitatively to the other experimental results, e.g. (−245, 217, −297, and 200 MHz) for the four Mn sites (37). Therefore, the inconsistency in the HFI constants suggests that the proposed methodology based on the comparison between calculated and experimentally measured HFI constants is not conclusive enough to determine the protonation state of the Mn_4_CaO_5_ cluster.

The spin structure of the low-spin S_2_ state has also been studied using pulsed electron–electron double resonance (PELDOR)/double electron–electron resonance (DEER) (39, 40). PELDOR can provide direct measurement of spin densities, by detecting a dipole interaction between tyrosine D radical (TyrD^•^) and the spin densities on each Mn site of the Mn_4_CaO_5_ cluster. Thus, PELDOR can directly obtain the spin densities, enabling a straightforward comparison with the spin densities calculated using the PSII structure.

PELDOR studies indicated a spin configuration of (↑↓↑↓) for (Mn1, Mn2, Mn3, and Mn4), with Mn1 having a large positive spin projection (*ρ*_1_ = 1.97), Mn3 having a small positive spin projection (*ρ*_3_ = 1.19), and Mn2 and Mn4 having negative spin projections (*ρ*_2_ = −1.2 and *ρ*_4_ = −0.96) (39). Consistently, Stich et al. (41) also reported a large spin projection for Mn1 with the D1-His332 ligand (*ρ*_1_ = 1.77) using the electron spin-echo envelope modulation (ESEEM). The large spin on Mn1 suggested in PELDOR (39) and ESEEM (41) studies was consistent with the ENDOR (37) results, but not with the previous calculations of HFI constants in the absence of the PSII protein environment (34, 36). As calculations of the HFI constants suffer from the uncertainty (29, 35, 37), the comparison between the spin projection distribution suggested in PELDOR and ESEEM studies and that calculated in theoretical models is more likely to provide further insights into the relevant spin structure of the Mn_4_CaO_5_ cluster in the PSII protein environment. Thus, the protonation state of the Mn_4_CaO_5_ cluster in the open-cubane S_2_ conformation corresponding to the *g* = 2 multiline signal has not been identified unambiguously, despite the extensive EPR studies including ENDOR, PELDOR, and ESEEM. Moreover, most theoretical calculations used to interpret spectroscopic results were conducted in the absence of the PSII protein environment (5, 29, 34, 37), which may be a reason for the uncertainty in the protonation state of the Mn_4_CaO_5_ cluster.

Here, we investigate the origin of the *g* = 2 multiline signal, using a quantum mechanical/molecular mechanical (QM/MM) approach and considering interactions between the open-cubane S_2_ conformation and the PSII protein environment.

## Methods

### Atomic coordinates

The X-ray diffraction structure of PSII monomer unit “A” (PDB code: 3ARC; 1.9-Å structure) (1) was used in the present study. It is worth noting that the 1.9-Å structure corresponds to S_1_ (1). Although the X-ray diffraction structure might have experienced over-reduction during data collection, leading to elongated Mn–O bonds (42–48), Suga et al. (2) reported that no significant structural difference exists between the XFEL structure for S_1_ and the 1.9-Å structure. The S_2_-state structure, obtained from the single-flash-minus-dark isomorphous difference Fourier map (1F-XFEL structure) at a slightly lower resolution (e.g. PDB code, 6JLK (9)), shows no significant differences compared to the 1.9-Å structure. Notably, the 1.9-Å structure contains more water molecules (1,442 molecules) than the S_2_-state structure (1,289 molecules). Furthermore, the calculated redox potential values are also essentially the same for the 1F-XFEL and/or 1.9-Å structure (49). Additionally, the *g* = 4.1 signal for the closed-cubane S_2_ conformation was also investigated using the 1.9-Å structures (28). To ensure consistency in the protein electrostatic environment for the QM/MM calculations and enable direct comparisons, the 1.9-Å structure was used for the open-cubane S_2_ conformation in the present study. Water molecules, protonation state of titratable residues, and atomic partial charges were treated as done in previous studies (28).

### QM/MM calculations

The unrestricted DFT method employing the B3LYP functional and LACVP* basis sets (Mn and Ca atoms: LANL2DZ [double ζ quality basis set with the Los Alamos effective core potential]; other atoms: and 6-31G*) (50) was used with the QSite (51) program as done in the previous study (28). The QM region was identical to that used for the closed-cubane S_2_ conformation (28). See Supplementary material for the atomic coordinates of the QM/MM-optimized geometry.

### Calculations of spin system

The exchange coupling values, *J_ij_*, between Mn(*i*) and Mn(*j*) (*i, j* = 1, 2, 3, 4, and *i* < *j*), were determined using the broken symmetry (BS) approach (6, 52, 53). Assuming the classical spin approximation, the total energies for distinct spin configurations can be described by the following equations (53):


(2) $${}^{13/2}E_{\left(↑↑↑↑\right)}=-6J_{12}\;-6J_{13}-6J_{14}\;-(9/2)J_{23}-(9/2)J_{24}-(9/2)J_{34},$$

(3) $${}^{7/2}E_{\left(↑↑↓↑\right)}=-6J_{12}+6J_{13}-6J_{14}+(9/2)J_{23}-(9/2)J_{24}+(9/2)J_{34},$$

(4) $${}^{7/2}E_{\left(↑↓↑↑\right)}=6J_{12}-6J_{13}-6J_{14}\;+(9/2)J_{23}+(9/2)J_{24}-(9/2)J_{34},$$

(5) $${}^{7/2}E_{\left(↑↑↑↓\right)}=\;-6J_{12}-6J_{13}+6J_{14}-(9/2)J_{23}+(9/2)J_{24}+(9/2)J_{34},$$

(6) $${}^{5/2}E_{\left(↓↑↑↑\right)}=6J_{12}+6J_{13}+6J_{14}-(9/2)J_{23}-(9/2)J_{24}-(9/2)J_{34},$$

(7) $${}^{1/2}E_{\left(↑↑↓↓\right)}=-6J_{12}+6J_{13}+6J_{14}+(9/2)J_{23}+(9/2)J_{24}-(9/2)J_{34}$$

(8) $${}^{1/2}E_{\left(↑↓↑↓\right)}=6J_{12}\;-6J_{13}+6J_{14}\;+(9/2)J_{23}-(9/2)J_{24}+(9/2)J_{34},$$

(9) $${}^{1/2}E_{\left(↑↓↓↑\right)}=6J_{12}+6J_{13}-6J_{14}-(9/2)J_{23}+(9/2)J_{24}+(9/2)J_{34},$$

where *^S^E*_sc_ is the total energy of the system for a given total spin *S*, obtained in QM/MM calculations (Table S1). The spin configuration sc refers to the configuration of spins for (Mn1, Mn2, Mn3, and Mn4), and *J_ij_* is the exchange coupling between Mn(*i*) and Mn(*j*). The pairwise *J* values were determined by solving the linear equations (Eqs. 1–8) using singular value decomposition to obtain the best solution in terms of the least-squares sense (52). Using the QM/MM-optimized geometries for all possible spin configurations, the total energy was calculated based on the adiabatic approximation (53).

The effective Hamiltonian describing the spin state of the Mn_4_CaO_5_ cluster can be expressed as follows:


(10) $$\hat{H}=\overset{4}{\underset{i=1}{\sum }}\;\beta {\hat{S}}_{i}\cdot g_{i}\cdot B_{0}\;+\;\sum {\hat{I}}_{i}\cdot A_{i}\cdot {\hat{S}}_{i}+{\hat{H}}_{\mathrm{ZFS}}+{\hat{H}}_{\mathrm{ex}}$$

where ${\hat{S}}_{i}$ is the operator for electron spin, ${\hat{I}}_{i}$ is the operator for nuclear spin, the ***g_i_*** value is the *g*-tensor, and ***A_i_*** is the effective hyperfine tensor in Mn(*i*). *β* is the Bohr magneton. In the present study, the ***g_i_*** value was approximated to be isotropic and independent of Mn(*i*), with a value of 2. ${\hat{H}}_{\mathrm{ZFS}}$ is the Hamiltonians of ZFS. ${\hat{H}}_{\mathrm{ex}}$ is the Hamiltonians of exchange interactions. The $\;{\hat{H}}_{\mathrm{ex}}\;$ term is expressed as


(11) $${\hat{H}}_{\mathrm{ex}}=-\underset{i<j}{\sum }\;2J_{ij}{\hat{S}}_{i}\cdot {\hat{S}}_{j}\;.$$

Because the ZFS parameters are unknown, ${\hat{H}}_{\mathrm{ZFS}}$ was neglected as done in the previous study (36). Here, Mn2, Mn3, and Mn4 are Mn(IV) (*S*_1_ = *S*_2_*= S*_3_ = 3/2) and Mn1 is Mn(III) (*S*_4_ = 2). By diagonalizing $\hat{H}$, the eigenenergy *E_n_*(***B*_0_**) of the *n*-th state |*n*(***B*_0_**)> was determined as a function of ***B*_0_**, excluding the hyperfine splitting term (54). The *n*-th excited energy *E_n_* (*n* = 0 for the ground state) in the manifold spin states is obtained from *E_n_*(***B*_0_** = 0).

The spin projection *ρ_i_* for Mn(*i*) was calculated as


(12) $$\rho_{i}=\;\frac{\left\langle {\hat{S}}_{i}\cdot {\hat{S}}_{t}\right\rangle }{\left\langle {\hat{S}}_{t}^{2}\right\rangle },$$

where


(13) $${\hat{S}}_{t}=\overset{4}{\underset{n=1}{\sum }}\;{\hat{S}}_{n}$$

is the total spin operator and $\hat{\left\langle A\right\rangle }$ in Eq. 12 represents the expectation value of *A*.

### PELDOR simulations

To simulate PELDOR measurements, the PELDOR result obtained from the previous experiments was used (39). PELDOR simulations were performed as done in the previous study (39). The signal amplitude *X*(*τ*′) depends on the time interval *τ*′ between the first and second pulses and can be expressed as follows:


(14) $$X(\tau^{\prime })\propto 1-p[1-\cos(2\pi D\tau^{\prime })]$$

where *p* is the fraction of spin affected by the pumping pulse, and *D* is the dipole interaction between the two spins. The expression for *D* between the spin density distributions of TyrD^•^ and the Mn_4_CaO_5_ cluster is given by:


(15) $$D=\underset{i,j}{\sum }\;\rho_{i}\rho_{j}\frac{g_{1}g_{2}\beta }{hR_{ij}^{3}}(1-3\mathrm{co}s^{2}\Theta_{ij})$$

where *ρ_i_* is the spin projection at the *i*-th (*i* = 1–7) carbon/oxygen atom of the TyrD^•^ molecule and *ρ_j_* is the spin projection at Mn(*j*). *R_ij_* is the distance between the *i*-th (*i* = 1–7) carbon/oxygen atom of the TyrD^•^ and Mn(*j*). *h* is the Planck constant, Θ*_ij_* is the angle formed between the external magnetic field ***H*** and the distance vector ***R_ij_***. *g*_1_ and *g*_2_ are *g*-factors and were assumed to be 2.00, neglecting *ρ* anisotropy as a first-order approximation (37).The signal amplitude *I*(*τ′*) is calculated by integrating over all angles and can be expressed as:


(16) $$I(\tau^{\prime })=\;\iint \;X(\tau^{\prime })\sin\theta d\theta d\varphi .$$

## Results and discussion

**Fig. 2.**
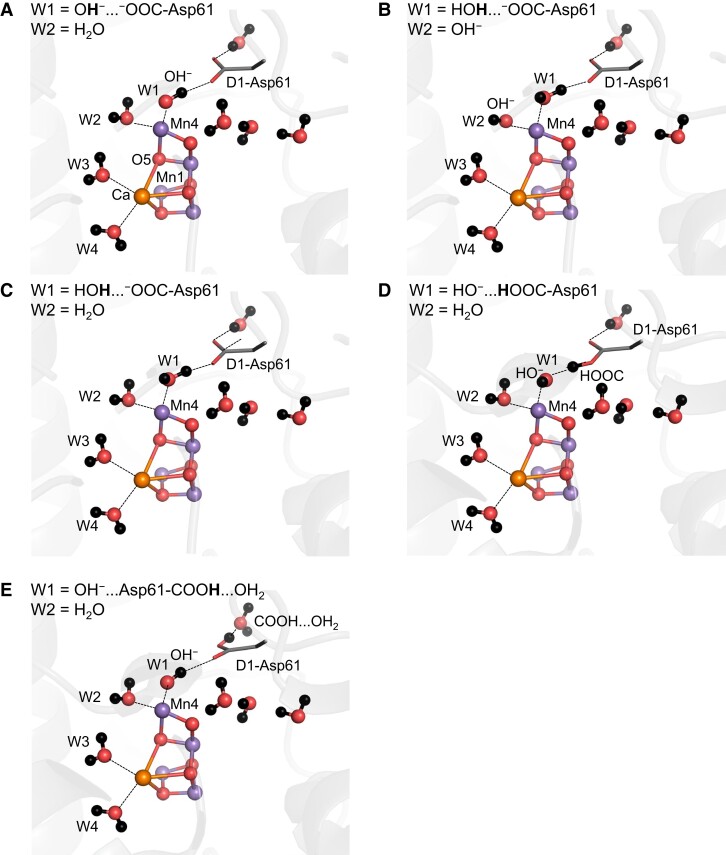
QM/MM-optimized geometries for the open-cubane S_2_ conformation. (A) W1 = OH^−^ and W2 = H_2_O. (B) W1 = H_2_O and W2 = OH^−^. (C) W1 = H_2_O and W2 = H_2_O. (D) W1 = HO^−^…HOOC-Asp61 and W2 = H_2_O. (E) W1 = OH^−^…Asp61-COOH…OH_2_ and W2 = H_2_O.

The Mn4 site has two water ligand molecules, W1 and W2. Ames et al. performed DFT calculations without considering the PSII protein environment to interpret the EPR results and proposed that W2 = OH^−^ (5, 6, 30). However, W1 forms a low-barrier H-bond with D1-Asp61 in the open-cubane S_2_ conformation (15–17, 19), whereas W2 only interacts with water molecules. Thus, the release of W1 toward D1-Asp61 can occur easily with respect to deprotonation of W2. Once the protonated side-chain of D1-Asp61 is reoriented (19, 55), the proton is further transferred toward the lumenal bulk surface. Based on these observations, the following five protonation states are investigated in the open-cubane S_2_ conformation: (i) W1 = OH^−^ and W2 = H_2_O; (ii) W1 = H_2_O and W2 = OH^−^; (iii) W1 = H_2_O and W2 = H_2_O; (iv) W1 = HO^−^…HOOC-Asp61 and W2 = H_2_O; and (v) W1 = OH^−^…Asp61-COOH…OH_2_ and W2 = H_2_O (Fig. 2 and Table 1).

**Table 1.** Calculated values for exchanging coupling *J_ij_* (cm^−1^) and the first excited state energy Δ*E*_01_ (i.e. energy difference between the ground and first-excited states) (cm^−1^) for the open-cubane S_2_ conformation.

Conformation/sample	*J*_12_	*J*_13_	*J*_14_	*J*_23_	*J*_24_	*J*_34_	*m_S_* ^total^ ^a^	Δ*E*_01_^b^
**This study**								
W1 = OH^−^ W2 = H_2_O	−33.0	9.1	0.5	16.1	2.2	−17.6	1/2	33.8
W1 = H_2_O W2 = OH^−^	−34.9	−2.3	1.5	11.7	1.5	−28.1	1/2	56.6
W1 = HO**H**…OOC-Asp61^c^ W2 = H_2_O	−28.8	−1.0	0.6	12.9	−0.7	−30.2	1/2	53.5
W1 = HO^−^…**H**OOC-Asp61^d^ W2 = H_2_O	−31.8	−1.2	1.5	18.8	4.2	−21.9	1/2	44.1
W1 = OH^−^…Asp61-COO**H**…OH_2_^e^ W2 = H_2_O	−31.8	4.4	0.6	16.7	1.8	−21.6	1/2	40.8
**Experiments**								
*Thermosynechococcus elongatus* PSII								22.4^e^
spinach PSII								24.7^e^, 36.5^f^

a The total magnetic spin quantum number *m_S_* at the ground state in the QM/MM calculation (see Table S1 for the calculated energies).

b Energy difference between the ground and the first-excited states obtained from the diagonalization of the spin Hamiltonian.

c The H^+^ is more populated at the W1 moiety along the low-barrier H-bond between W1 and D1-Asp61.

d H^+^ is more populated at the D1-Asp61 moiety along the low-barrier H-bond between W1 and D1-Asp61.

e Protonated D1-Asp61 donates an H-bond to a water molecule in the D1-Glu65/D2-Glu312 channel.

e Ref. (34).

f Ref. (33).

### Exchange couplings

The exchange coupling values *J_ij_* were calculated using the five QM/MM-optimized geometries shown in Fig. 2. Regardless of the protonation states of W1 and W2, the exchange couplings between Mn1 and Mn2 (*J*_12_) and Mn3 and Mn4 (*J*_34_) are consistently negative and have large-magnitude values (−18 to −35 cm^−1^) in all cases. When W1 = OH^−^ and W2 = H_2_O, the first excited state energy Δ*E*_01_ (34 cm^−1^) is most consistent with the observed value (22–37 cm^−1^) (33, 34) (Table 1).

As the negative couplings of *J*_12_ and *J*_34_ imply that the Mn1/Mn2 and Mn3/Mn4 pairs favor opposite spin directions to each other, the spin configurations of (↑↓↑↓) and (↑↓↓↑) for Mn1(III)Mn2(IV)Mn3(IV)Mn4(IV) [the magnetic spin quantum numbers (*m*_S1_, *m*_S2_, *m*_S3_, *m*_S4_) = (4/2, −3/2, −3/2, 3/2), (4/2, −3/2, 3/2, −3/2) and the total magnetic spin quantum number *m_S_*^total^ = 1/2] have the lowest and second-lowest energies, respectively (Table S1). Indeed, in all conformations, the calculated spin projections indicate the spin configuration of (↑↓↑↓) or (↑↓↓↑) in the ground state of the spin Hamiltonian (Table 2). The (↑↓↑↓) configuration exhibits in the ground state only when W1 = OH^−^ and W2 = H_2_O, which reflects the fact that the difference in the BS energy *^S^E*_sc_ between the (4/2, −3/2, −3/2, 3/2) and (4/2, −3/2, 3/2, −3/2) conformations is small (21 cm^−1^) with respect to the magnitude of the *J* couplings (Table S1). The (↑↓↑↓) configuration with W1 = OH^−^ and W2 = H_2_O is consistent with that observed in PELDOR studies (39). Thus, the open-cubane S_2_ conformation is most likely with W1 = OH^−^ and W2 = H_2_O. Note that the protonation state with W1 = OH^−^ and W2 = H_2_O was also reported for the closed-cubane S_2_ conformation for the *g* = 4.1 signal (28).

**Table 2.** Calculated spin projections *ρ*_1_, *ρ*_2_, *ρ*_3_, and *ρ*_4_ for Mn1, Mn2, Mn3, and Mn4 in the ground state of the diagonalized spin Hamiltonian for the open-cubane S_2_ conformation.

Conformation	*ρ*_1_	*ρ*_2_	*ρ*_3_	*ρ*_4_	Spin configuration (Mn1, Mn2, Mn3, Mn4)
W1 = OH^−^ W2 = H_2_O	1.818	−0.857	0.512	−0.473	(↑↓↑↓)
W1 = H_2_O W2 = OH^−^	1.722	−0.941	−0.886	1.105	(↑↓↓↑)
W1 = HO**H**…OOC-Asp61^a^ W2 = H_2_O	1.849	−0.986	−0.809	0.946	(↑↓↓↑)
W1 = HO^−^…**H**OOC-Asp61^b^ W2 = H_2_O	1.555	−0.890	−0.972	1.307	(↑↓↓↑)
W1 = OH^−^…Asp61-COO**H**…OH_2_^c^ W2 = H_2_O	1.958	−1.000	−0.565	0.606	(↑↓↓↑)

a The H^+^ is more populated at the W1 moiety along the low-barrier H-bond between W1 and D1-Asp61.

b The H^+^ is more populated at the D1-Asp61 moiety along the low-barrier H-bond between W1 and D1-Asp61.

c Protonated D1-Asp61 donates an H-bond to a water molecule in the D1-Glu65/D2-Glu312 channel.

### PELDOR

The values of the spin projection *ρ* in the ground state of the spin Hamiltonian of Eq. (12) were calculated based on the five QM/MM-optimized geometries (Fig. 2). Although the calculated *ρ* values depend on the protonation states of W1 and W2, *ρ*_1_ is the largest among the four *ρ_i_* values for all protonation states (Table 2).

**Fig. 3.**
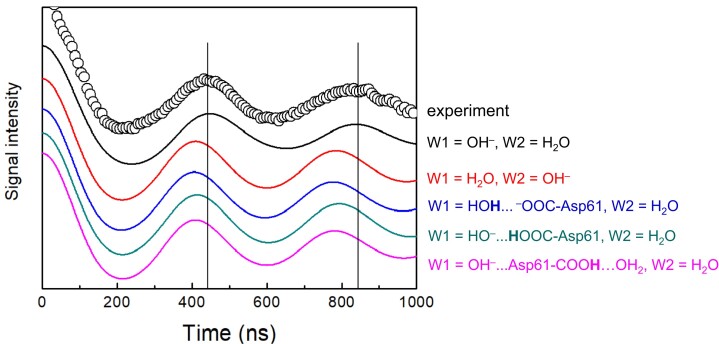
Simulated PELDOR signals arising from the interaction between TyrD^•^ and the Mn_4_CaO_5_ cluster in the high-spin S_2_ state. The experimental signals for spinach PSII (39) are shown by circles. The vertical lines indicate the peak positions in the experimental signal.

The PELDOR signal was simulated using the calculated *ρ* values (Fig. 3). The experimentally observed oscillation pattern (39) is best reproduced when W1 = OH^−^ and W2 = H_2_O in the QM/MM calculation. In contrast, the observed pattern is not reproduced in the other protonation states as the frequencies in the calculated signals are shifted faster (i.e. the peak positions in the calculated signals are shifted). These results suggest that W1 = OH^−^ and W2 = H_2_O are the protonation state for the low-spin state of the open-cubane S_2_ conformation.

When W1 = OH^−^ and W2 = H_2_O, the calculated *ρ*_1_ value for Mn1(III) is 1.82 (Table 2), which is consistent with *ρ*_1_ ≈ 2 suggested in previous PELDOR studies (39) and *ρ*_1_ = 1.7 suggested in ENDOR and ESEEM studies (37, 41). All of these studies show that *ρ*_1_ has the largest magnitude among the four *ρ_i_* values (Table 2), including DFT calculations of the Mn_4_CaO_5_ cluster performed by Ames et al. (5) in the absence of the PSII protein environment.

### Uncertainty in the calculated *A_i,_*_iso_ values

Ames et al. (5) performed DFT calculations without considering the PSII protein environment and found that the magnitude of the calculated *ρ*_1_ value for Mn1(III) was the largest among those for the four Mn sites in the open-cubane S_2_ conformation. This result is consistent with ESEEM (41), PELDOR (39), and the present calculation (Table 2).

While the *ρ_i_* value is already known and the *a_i,_*_iso_ value can be calculated using the BS approach (52), if Eq. 1 was relevant to the Mn_4_CaO_5_ cluster, all these studies would indicate that *A_i,_*_iso_ was largest at Mn1(III) among the four Mn sites. However, in the same study, Ames et al. (5) controversially reported that the calculated *A_i,_*_iso_ value was the largest at Mn4(IV). This inconsistency between experimentally measured *A_i,_*_iso_ values (29, 37) and calculated *A_i,_*_iso_ values (5) suggests that Eq. 1 is unlikely to be applicable to the Mn_4_CaO_5_ cluster.

The source of the inconsistency may be due to insufficient consideration of anisotropy in Eq. 1. Eq. 1 is *guaranteed only in the absence of ZFS* (36) and may be applicable to the two-spin system in a model compound with the *a_i,_*_iso_ values obtained using the BS calculation (38). However, it seems unlikely that Eq. 1 is directly applicable to the multi-coupled system due to its anisotropy. In particular, inter-dipole interactions cause the anisotropy in the Mn_4_CaO_5_ cluster, because the total ZFS consists of ZFSs on the four Mn sites (56, 57). Therefore, a direct comparison between *A_i,_*_iso_ converted using Eq. 1 from calculated *ρ_i_* values and those measured experimentally has not been established in the Mn_4_CaO_5_ cluster (e.g. (29, 37)).

### Formation of OH^−^ at W1 in EPR-detected S_2_ in EPR experiments

**Fig. 4.**
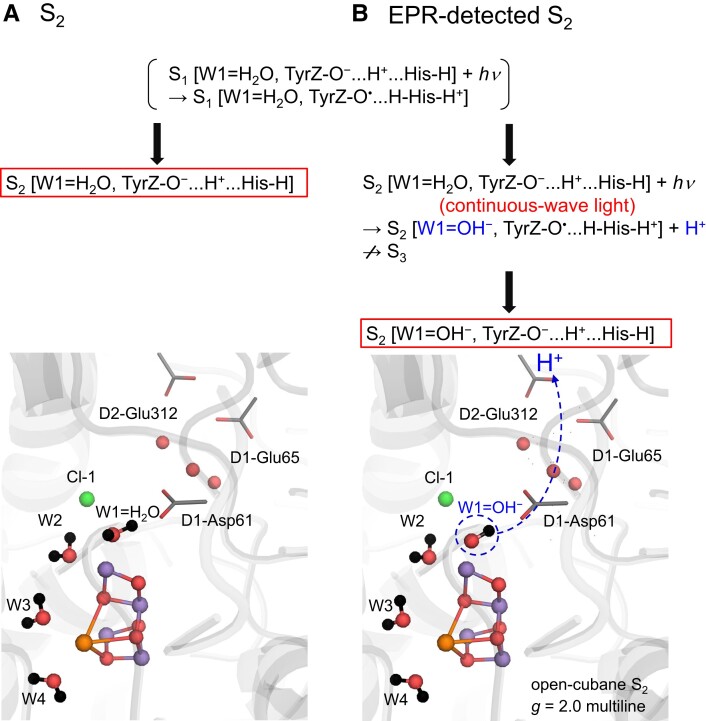
Comparison of the protonation states of the open-cubane S_2_ structure in (A) S_2_ (W1 = H_2_O) and (B) EPR-detected S_2_ (W1 = OH^−^) exposed to continuous-wave light for ∼5 minutes at low temperature (200 K). While the S_2_ to S_3_ transition is blocked, proton release from W1 can still occur, leading to the formation of OH^−^ at W1 in the EPR-detected S_2_ sample. Boxed states indicate resulting states.

H_2_O at W1 releases the proton during the S_2_ to S_3_ transition (19, 55). In the actual S_2_ state, the proton migrates along the low-barrier H-bond between W1 and D1-Asp61, as demonstrated in Fourier transform infrared spectroscopy (16) and theoretical (19, 55) studies. However, at this stage, the proton is not yet released toward the lumenal bulk surface (e.g. (15, 58)) (Fig. 4A). In contrast, the present results show that the open-cubane S_2_ conformation with W1 = OH^−^ and W2 = H_2_O is most consistent with the observed first excited energy Δ*E*_01_ in *T*_1_ measurements (33, 34, 59) (Table 1) and the observed PELDOR signal (39) (Fig. 3) for the low-spin S_2_ state. That is, OH^−^ already exists at W1 in the EPR-detected S_2_ samples.

The difference in the protonation state between S_2_ and EPR-detected S_2_ could be due to the difference in the precursor. Although charge separation occurs in S_2_, S_2_ does not proceed to S_3_ at low temperature (∼200 K) in EPR measurements (60, 61). However, the sample is under continuous-wave light conditions, which still allows P680 to be photoexcited, oxidizing [TyrZ-O^−^…H ^+^ …N-His190-NH] to [TyrZ-O^•^…HN-His190-NH]^+^ and deprotonating the lowest-p*K*_a_ site at the Mn_4_CaO_5_ moiety in S_2_ (e.g. (18, 62)). As QM/MM calculations performed in the presence of the PSII protein environment have suggested that W1 is the lowest-p*K*_a_ site among all titratable sites at the Mn_4_CaO_5_ moiety in S_2_ (63), it seems possible that W1 releases the proton, forming OH^−^ under continuous-wave light conditions in EPR measurements (Fig. 4B). This may explain the discrepancy between S_2_ and EPR-detected S_2_.

## Conclusions

The exchange coupling *J_ij_* calculated in the presence of the PSII protein environment indicates that the spin configuration of the open-cubane S_2_ conformation is (↑↓↑↓) or (↑↓↓↑) for Mn1(III)Mn2(IV)Mn3(IV)Mn4(IV) (Table 1). Diagonalization of the spin Hamiltonian obtained using the *J* couplings shows that when W1 = OH^−^ and W2 = H_2_O, the first excited energy (Δ*E*_01_ = 34 cm^−1^) in the manifold spin states is consistent with the experimentally observed value (27–37 cm^−1^) (Table 1). The magnitude of the calculated *ρ*_1_ value for Mn1(III) is the largest among those for the four Mn sites in the open-cubane S_2_ conformation (Table 2), which is consistent with ESEEM (41) and PELDOR (39) studies and previous DFT calculations conducted without considering the PSII protein environment (5). The PELDOR signal observed for the low-spin S_2_ state (39) is reproduced only when W1 = OH^−^ and W2 = H_2_O in the open-cubane S_2_ conformation (Fig. 3). These results obtained from the present QM/MM calculations conducted in the presence of the PSII protein environment consistently suggest that the *g* = 2 multiline signal in EPR corresponds to the open-cubane S_2_ conformation with W1 = OH^−^ and W2 = H_2_O (Fig. 4B).

## Supplementary Material

pgad244_Supplementary_Data

## Data Availability

All data are included in the manuscript and/or supporting information.
