# Supplementary material for: Insights into the protonation state and spin structure for the g = 2 multiline electron paramagnetic resonance signal of the oxygen-evolving complex
Source: PNAS Nexus. 2023 Jul 28;2(8):pgad244. doi: 10.1093/pnasnexus/pgad244 (PMC10411963; doi:10.1093/pnasnexus/pgad244)
Supplement: pgad244_Supplementary_Data [file pgad244_supplementary_data.zip › EPR_open_cubane-SI_2.1_HI_unmarkedKS.pdf]

Supporting information for:

Insights into the protonation state and spin structure for the *g*=2 multiline EPR signal of the oxygen-evolving complex

Keisuke Saito [1,2]*, Shunya Nishio [1], Mizue Asada [4], Hiroyuki Mino [3], Hiroshi Ishikita [1,2]*

1) Department of Applied Chemistry, The University of Tokyo, 7-3-1 Hongo, Bunkyo-ku, Tokyo 113-8654, Japan

2) Research Center for Advanced Science and Technology, The University of Tokyo, 4-6-1 Komaba, Meguro-ku, Tokyo 153-8904, Japan

3) Division of Material Science, Graduate School of Science, Nagoya University, Furo-cho, Chikusa-ku, 464-8602 Nagoya, Aichi, Japan

4) Institute for Molecular Science, 38 Nishigo-Naka, Myodaiji, Okazaki, 444-8585, Japan.

CORRESPONDING AUTHOR:

Keisuke Saito, **E-mail:** ksaito@appchem.t.u-tokyo.ac.jp

Hiroshi Ishikita, **E-mail:** hiro@appchem.t.u-tokyo.ac.jp

Research Center for Advanced Science and Technology, The University of Tokyo, 4-6-1 Komaba, Meguro-ku, Tokyo 153-8904, Japan, Tel. +81-3-5452-5056, Fax. +81-3-5452-5083,

Table S1. Adiabatic total energy ${}^{S}E_{sc}$ (cm^{-1}) in the broken symmetry calculation using the optimized geometries of all possible spin configurations.

**W1**			**OH^-**	**H_2O**	**H_2O**	**HO^-...HOOC-Asp61**	**OH^-...Asp61-COOH...OH_2**
**W2**			**H_2O**	**OH^-**	**H_2O**	**H_2O**	**H_2O**
**m_s^{total}**	**(m_{s1}, m_{s2}, m_{s3}, m_{s4})**	**Spin configuration[a]**					
13/2	(4/2, 3/2, 3/2, 3/2)	(↑↑↑↑)	425	686	636	555	507
7/2	(4/2, 3/2, −3/2, 3/2)	(↑↑↓↑)	523	460	437	518	515
7/2	(4/2, −3/2, 3/2, 3/2)	(↑↓↑↑)	197	335	369	386	292
7/2	(4/2, 3/2, 3/2, −3/2)	(↑↑↑↓)	295	415	334	418	336
5/2	(−4/2, 3/2, 3/2, 3/2)	(↓↑↑↑)	147	207	253	182	187
1/2	(4/2, 3/2, −3/2, −3/2)	(↑↑↓↓)	705	795	741	766	793
1/2	(4/2, −3/2, 3/2, −3/2)	(↑↓↑↓)	21	137	142	163	89
1/2	(4/2, −3/2, −3/2, 3/2)	(↑↓↓↑)	0	0	0	0	0

[a] (Mn1, Mn2, Mn3. Mn4).
